# Bile acids modulate reinstatement of cocaine conditioned place preference and accumbal dopamine dynamics without compromising appetitive learning

**DOI:** 10.1038/s41598-023-40456-3

**Published:** 2023-08-17

**Authors:** Daniele Zanella, Nicholas K. Smith, J. Andrew Hardaway, Anna Marie Buchanan, Clarence H. Mullins, Aurelio Galli, Angela M. Carter

**Affiliations:** 1 Department of Surgery, Heersink School of Medicine, University of Alabama at Birmingham, Birmingham, USA; 2 Department of Biology, University of Pennsylvania, Philadelphia, USA; 3 Department of Psychiatry and Behavioral Neurobiology, Heersink School of Medicine, University of Alabama at Birmingham, Birmingham, USA; 4 Center for Inter-Systemic Networks and Enteric Medical Advances (UAB CINEMA), Birmingham, USA

**Keywords:** Reward, Feeding behaviour

## Abstract

Psychostimulants target the dopamine transporter (DAT) to elicit their psychomotor actions. Bile acids (BAs) can also bind to DAT and reduce behavioral responses to cocaine, suggesting a potential therapeutic application of BAs in psychostimulant use disorder. Here, we investigate the potential of BAs to decrease drug-primed reinstatement when administered during an abstinence phase. To do this, after successful development of cocaine-associated contextual place preference (cocaine CPP), cocaine administration was terminated, and animals treated with vehicle or obeticholic acid (OCA). When preference for the cocaine-associated context was extinguished, mice were challenged with a single priming dose of cocaine, and reinstatement of cocaine-associated contextual preference was measured. Animals treated with OCA demonstrate a significantly lower reinstatement for cocaine CPP. OCA also impairs the ability of cocaine to reduce the clearance rate of electrically stimulated dopamine release and diminishes the area under the curve (AUC) observed with amperometry. Furthermore, the AUC of the amperometric signal positively correlates with the reinstatement index. Using operant feeding devices, we demonstrate that OCA has no effect on contextual learning or motivation for natural rewards. These data highlight OCA as a potential therapeutic for cocaine use disorder.

## Introduction

The dopaminergic system is a major target of psychostimulants such as amphetamines and cocaine. These substances elicit their psychomotor actions by targeting the dopamine (DA) transporter (DAT), a presynaptic membrane protein responsible for the reuptake of DA from the synaptic cleft following vesicular release^1^. Cocaine acts as a competitive inhibitor of DA uptake, leading to an increase in extracellular levels of DA^2,3^. This increase in DA levels dysregulates both DA neurotransmission and the activity of downstream neurons. One of the more relevant dopaminergic circuits, in relation to psychostimulants and the development of substance use disorders, is the mesolimbic pathway, i.e. the projections of ventral tegmental area (VTA) neurons to the nucleus accumbens (NAc)^4^.

Psychostimulant use disorder (PUD) is one of the leading causes of overdose in the United States, second only to opioids (National Institute on Drug Abuse; National Institutes of Health; U.S. Department of Health and Human Services). Unfortunately, there are currently no approved medications to treat PUD. Therefore, identification of new molecular targets for the treatment of PUD is sorely needed. Recent studies demonstrate that bile acids (BAs) can interact directly with the DAT^5,6^ and regulate its electrical activity. This observation, combined with previous reports that BAs can reduce behavioral responses to cocaine^7^, suggests a potential application of BAs for the treatment of PUD.

BAs are important amphiphilic molecules, whose primary function is to act as emulsifiers for fat absorption^8,9^. However, additional functions as signaling molecules that influence multiple aspects of metabolism, immunity, and even cancer progression have now been uncovered^9–13^. A role for BAs in modulating brain function is also gaining broader acceptance, as studies have demonstrated an impact of BAs on neuroprotection^14–18^, food reward and motivation^13,19,20^, as well as behavioral responses to drugs of abuse^7^. To elicit these effects, BAs act as ligands for multiple cellular receptors, including the cell surface receptor Takeda G protein-coupled Receptor 5 (TGR5)^21,22^ and the nuclear receptor farnesoid X receptor (FXR)^23^.

Intriguingly, Gallbladder-to-Ileum anastomosis (GB-IL), a surgery designed to induce metabolic improvement in obesity models, leads to increased levels of circulating BAs^24^. This procedure also reduces cocaine-induced increases in DA levels in the NAc^7^. Consistently, cocaine-associated behaviors are also reduced, such as hyperlocomotion, locomotor sensitization and drug-associated contextual preference. These effects parallel those observed from feeding mice the semi-synthetic BA obeticholic acid (OCA)^7^.

Encountering relapse is an almost inevitable part of the recovery process of individuals with PUD^25,26^. Thus, development of pharmacological interventions for relapse prevention is critical for successfully treating PUD. A commonly used pre-clinical animal model of drug relapse is the drug priming-induced reinstatement of drug associated contextual preference (e.g. cocaine-induced condition place preference (cocaine CPP))^27^. Here, we demonstrate that OCA reduces drug priming-induced reinstatement for cocaine CPP. This reduction was associated with the ability of OCA to inhibit cocaine-induced increases in accumbal DA levels upon reinstatement.

## Materials and methods

### Chemicals and solutions

Obeticholic acid (OCA; or 6α-ethyl-chenodeoxycholic acid (6-ECDCA)) and (2-Hydroxypropyl)-beta-cyclodextrin were purchased from Fisher Scientific. Cocaine hydrochloride and injectable 0.9% NaCl solutions were purchased from Millipore-Sigma. NMDG-HEPES recovery solution (NMDG) had the following composition (in mM): 100 NMDG, 2.5 KCl, 1.2 NaH_2_PO_4_*H_2_O, 30 NaHCO_3_, 20 HEPES, 25 D-Glucose, 10 MgSO_4_*7H_2_O, 0.5 CaCl_2_*2 H_2_O, 5 L-Ascorbic Acid, 2 Thiourea, 3 Sodium Pyruvate, 12 *N*-Acetyl-l-Cysteine. Osmolarity ranged between 315 and 320 mOsm; pH 7.4. Artificial cerebrospinal fluid solution (aCSF) had the following composition (in mM): 125 NaCl, 2.5 KCl, 12 NaH_2_PO_4_*H_2_O, 1 MgCl_2_*6H_2_O, 2 CaCl_2_*2H_2_O, 26 NaHCO_3_, 10 D-Glucose. Osmolarity ranged between 315 and 320 mOsm; pH 7.4. Osmolarity was controlled using a Vapro 5520 (Wescor).

### Behavior

All animal work was performed in accordance with the Animal Welfare Act and the Guide for the Care and Use of Laboratory Animals under UAB Institutional Animal Care and Use Committee approved protocols. All animal experiments were performed and reported according to ARRIVE guidelines.

Male wild type C57Bl/6J mice, from The Jackson Laboratory, aged 6 to 12 weeks, were utilized for all experiments. Animals were subject to a 12 h light/dark cycle and had access to ad libitum water. Food access was ad libitum for the reinstatement experiments, regulated for the feeding experiments. Experiments were not blinded. To allow for gut bioavailability of OCA without the stress of oral gavage, OCA was administered to mice daily by voluntary oral consumption in jellies at 10 mg/kg BW^7^. OCA was initially dissolved in beta cyclodextrin (20% w/v), and then mixed within palatable jellies. Jellies were composed of gelatin (10% w/v), sucralose (18.5% w/v), and artificial strawberry flavoring (8% v/v) in water. Control jellies contained beta cyclodextrin without OCA. Jelly consumption was monitored daily.

#### Reinstatement experimental design

Conditioned Place Preference (CPP) was performed as previously described^7^, with minor modifications. Two-chamber apparati (MED-CPP2-3013-2, Med-Associates) with distinct rod and mesh floor inserts were used. Mice were acclimated in the testing room for at least 1.5 h each day, prior to testing. The testing timeframe was 20 min. On Day1 (D1), mice were placed into the rod floor side, with free access to both sides of the apparatus (Pre-Test). During D2–D9 (Conditioning), i.p. injections of either 20 mg/kg cocaine or saline were paired with placement in a specific side of the apparatus. The side determined “least preferred” during Pre-Test was paired with cocaine injections, while the “preferred” side was paired with saline. Mice received cocaine and saline on alternating days. On D10, mice were granted free access to both sides without injections (Post-Test). CPP Score was calculated as time spent in cocaine-paired side during Post-Test minus time spent in that side on Pre-Test, divided by time spent in the other side on Pre-Test. After Post-Test verified the development of CPP, mice were randomly (computer-based random order generator) divided into two groups to receive either vehicle or 10 mg/kg OCA treatment, which continued until termination of the experiment. Mice were allowed access to both sides of the apparatus during extinction testing. Successful extinction was defined as losing 50% or more of the preference for the cocaine-paired side. The day following successful extinction (Dx + 1), mice were injected with cocaine (20 mg/kg, i.p.) and reinstatement of CPP, expressed as Reinstatement Index (R.I.), was calculated as the time spent in the cocaine-paired compartment divided by the time spent in the same compartment the prior day (day of extinction, Dx).

#### Feeding experiments design

Home cages (One Cage Micro-Isolator) were set up with one FED3 device^28^ in each, as the only food source, with ISO pads (Braintree Scientific) for bedding. Standard chow diet, pelleted to fit the FED3 devices, was purchased from TestDiet. Mice were singly housed to allow assessment of individual food consumption and given unlimited access to the devices in their home cage. Animals were acclimated to the FED3 devices as their main food source, as well as to once daily jelly consumption, for 3 days prior to the start of experiments. Mice were administered OCA (10 mg/kg) or vehicle treatment once a day, via feeding of jellies, throughout the experiment. For 3 weeks, mice received their main food supply ad libitum, with the FED3 devices set on Free Feeding (devices automatically dispense a new pellet upon retrieval of the previous pellet). Under these free access conditions, some mice will hoard food, i.e. take high numbers of pellets, but not consume them. Hoarders can be identified by food accumulation on the floor of the home cage. Daily consumption numbers were excluded on days in which mice were found to be hoarding. On week 4, devices were switched to Fixed Ratio 1 (FR1), and mice learned to perform an action (nose poke on a designated port) to receive food. Each FED3 device had one designated port and one inactive port. An auditory cue and a visual cue were coupled with dispensing of the food pellet upon successful performance of the task. On week 5, devices were switched to Fixed Ratio 3 (FR3), increasing the number actions (nose pokes to designated port) required to receive a pellet to 3. Poke Efficiency for FR1 and FR3 was calculated as ratio between the number of correct pokes (designated port) and the total number of pokes (designated port plus inactive port). On the final day of the experiment, devices were switched to Progressive Ratio (PR) in which the number of actions required to obtain a food pellet continuously increases in non-linear fashion with every successfully completed task (1–2–4–6–9–12–15–20-etc.). Breakpoint is defined as the number of pokes in the last completed task within a 3-h threshold.

### Brain extraction and slice amperometry

Twelve mice (6 from each treatment group) were harvested after reinstatement. After brief isoflurane administration, cervical dislocation was performed, brains were extracted, and coronal slices (thickness 300 µm) were prepared in ice-cold aerated NMDG using a vibratome VT1000S (Leica). Slices containing the nucleus accumbens (NAc) were transferred for recovery into aerated (bubbling with 95% O_2_-5% CO_2_) NMDG at 32 °C for 12 min, and then transferred to aerated aCSF at 28 °C until use (4 h maximum for viability).

Slices were transferred to the electrophysiological rig. The flow rate was 1.5 mL/min. All solutions were individually aerated, and the temperature was held at 30 °C through a solution heater (SH-27B, Warner Instruments). The stimulation electrode (MS303/3-B/SPC ELECT SS 2C TW 0.0005, P1 Technologies), connected to digitally controlled pulse generator (Master8 with Iso-Flex stimulus isolator, A.M.P.I.), was placed first. The amperometric electrode, controlled by an amplifier (Axopatch 200B, Molecular Devices) and held at 400 mV, was then placed at a depth varying between 50 and 75 µm. Controlling software was Clampex 10.6 (Molecular Devices). Data collection was carried out with a recording protocol (sampling 1 kHz filtered at 100 Hz with a LPF-8, Warner Instruments) delivering a single current pulse of − 200 µA every 5 min. The protocol was designed to elicit release of DA from the slice while allowing the slice to recover after each stimulus. After a stable baseline in aCSF was achieved (3 recordings with peak variation within 10%), perfusion was switched to aCSF + 10 µM cocaine and up to 3 more recordings were taken. Data was digitized using a Digidata 1440A (Molecular Devices). Data analysis was performed with Clampfit 10.6. Traces were digitally filtered (Bessel 8-poles, 10 Hz).

### OCA detection in blood and brain tissue

Six experimentally naïve animals were fed jellies for voluntary consumption as described previously^7^. One animal received vehicle; five received 10 mg/kg OCA. Consumption of jellies was evaluated every 30 min until all material was eaten. Animals were fed once a day for 4 days, then euthanized approximately 3 h post introduction of the final jelly. Blood was immediately removed by retro-orbital bleeding and serum isolated. Cardiac perfusions were performed, following blood collection, with cold Phosphate Buffered Saline (PBS). Brains were extracted, and then a coronal brain matrix (RBM-2000C, ASI Instruments) was used to obtain 1 mm thick sections. These were further processed into 2 mm diameter punches containing NAc. All samples were flash frozen in liquid nitrogen and then sent to the Targeted Metabolomics and Proteomics Laboratory Core at UAB for OCA quantification by LC–MS/MS. Briefly, samples were extracted using the Bligh-Dyer Protocol and loaded onto a 2.1 × 50 mm, 2.6 μm Kinetex PS C_18_, 80 Å reverse-phase column (Phenomenex, Torrance, CA) equilibrated with 20% acetonitrile in 10 mM ammonium acetate, resolved using a linear gradient of 20–40% acetonitrile in 10 mM ammonium acetate, and analyzed by a 5600 Triple-TOF mass spectrometer. Spectra were centroided and de-isotoped by Analyst software, version 1.8 T and processed using PeakView Software 2.2.

### Statistical analysis

Statistical analysis and graph preparation was performed with GraphPad Prism software, version 6. The type of statistical test used for each experiment is specified in the figure legends. Statistical significance was defined as *p* < 0.05 for all tests. Data are presented as means ± standard error of the mean. All sample populations have been tested for normality using the Shapiro–Wilk test. The ROUT method (Q = 1%) was used as an outlier identification test and used as exclusion criteria.

## Results

### OCA treatment reduces cocaine reinstatement

**Figure 1:**
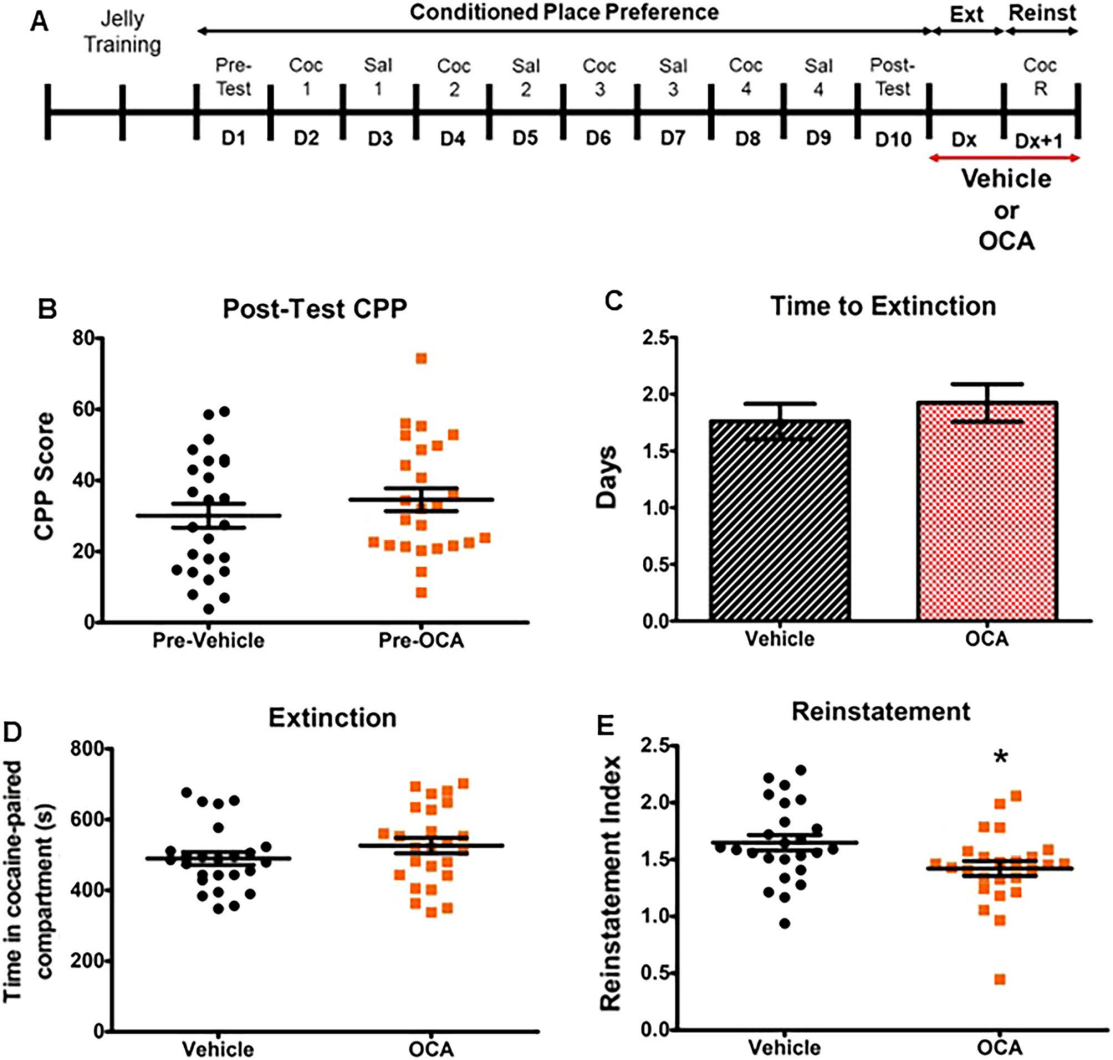
OCA inhibits cocaine priming-induced reinstatement for cocaine CPP. (**A**) Schematic of study design. Mice were acclimated to consuming control jellies for 2 days. Then, mice underwent a CPP protocol for cocaine (20 mg/kg, i.p.) from D1 to D10, as described in the Methods. After D10, the animals were orally administered either vehicle or OCA (10 mg/kg) in jellies. Mice were then evaluated daily for extinction (loss of half the CPP present at D10); the variable number of days that each animal takes to achieve extinction is expressed as Dx. The day after reaching extinction (Dx + 1), animals were exposed to a priming dose of cocaine (20 mg/kg, i.p.) and evaluated for reinstatement of cocaine CPP. (**B**) CPP score at D10 between randomly divided groups (two-tailed Student’s *t* test, *p* = 0.34, t = 0.96, n = 25). (**C**) Number of days to achieve extinction of cocaine CPP in mice receiving either vehicle or OCA (two-tailed Student’s *t* test, *p* = 0.49, t = 0.69, n = 25). (**D**) Time spent (out of 20 min total) in cocaine-paired compartment, on the day of extinction, in animals receiving either vehicle or OCA (two-tailed Student’s *t* test, *p* = 0.21, t = 1.28, n = 25). (**E**) Reinstatement index in mice treated with either vehicle or OCA and receiving a priming dose of cocaine (20 mg/kg, i.p.) (two-tailed Student’s *t* test with Welch’s correction, *p* = 0.02, t = 2.41, n = 25).

An increase in circulating BAs impairs cocaine-induced locomotor sensitization, as well as cocaine-associated contextual preference (cocaine conditioned place preference, (CPP))^7^. However, it is not clear whether BAs (i.e. OCA) can impair cocaine priming-induced reinstatement (reinstatement) of cocaine CPP, a commonly used pre-clinical animal model of drug relapse^27^. To test this, mice were first trained in a two-chamber behavioral apparatus using a traditional cocaine CPP paradigm^7^. In this paradigm, injection of cocaine (20 mg/kg, i.p.) is coupled with placement into a specific distinct chamber, while injection of saline is coupled to placement in a second distinct chamber, each performed on alternating days, for 8 days (Fig. 1A). After verifying the expression of cocaine contextual preference, mice were randomly assigned to receive either vehicle or OCA, which continued until termination of the experiment. Vehicle and OCA were administered daily by voluntary oral consumption of jellies (see “Methods”). The two randomly assigned groups of mice (Pre-Vehicle; Pre-OCA) showed no significant difference in cocaine CPP scores (Fig. 1B). Mice were then allowed to undergo extinction, defined by a decrease of at least 50% of initial cocaine CPP scores. The average time required to reach extinction was not significantly different between treatment groups (Fig. 1C), and the time spent in cocaine-paired compartment on the last day of extinction was also similar between groups (Fig. 1D). Mice were then tested for reinstatement of cocaine-associated contextual preference by administration of a single injection of cocaine (20 mg/kg, i.p.) and free access to either chamber. Notably, animals that received OCA showed a significantly lower reinstatement index (R.I. = 1.42 ± 0.07) compared to animals that received vehicle (Fig. 1E, R, I. = 1.65 ± 0.07).

To confirm these effects are potentially driven by OCA action in the brain, we verified the ability of OCA to be absorbed, through the gastrointestinal track, into the blood and NAc brain tissue. To do this, we fed six animals jellies, as described above, and performed LC–MS/MS analysis of serum and NAc tissue. One animal received control jellies and five animals received OCA-containing jellies (10 mg/kg). OCA is a synthetic bile acid, and therefore, not present in untreated animals. Lack of co-migration with naturally occurring bile acids was confirmed using samples from the control animal and standard solutions of OCA. Analyses of serum samples demonstrated clear absorption of OCA into the blood of all animals (Supplementary Fig. 1A, *bottom*). Total quantified values varied, possibly due, at least in part, to differences in consumption rates (Supplemental Fig. 1A, *top*) which would shift the phase of the pharmacokinetic window each animal was within at harvest. In addition to the parent OCA molecule, MS analysis detected robust signals for taurine-conjugated OCA (T-OCA) in the serum of treated animals. This is a bile acid salt, formed in the liver after OCA is reabsorbed from the blood by the enterohepatic circulation^29^. The amount of OCA that transitions into the brain is lower than levels achieved in the blood. However, in the three mice that exhibited the highest serum levels of OCA (M2, M3, and M4), we were able to detect OCA in the NAc as well (Supplemental Fig. 1B). Previously, it has been shown, in rats, that bile acids in the plasma accumulate in the brain and their concentration correlates with the serum levels^30^. We have also shown, in mice, the accumulation of the bile acid taurocholic acid, in the brain, using timed harvest following direct venous injection^7^. Together, these data demonstrate that bile acids can cross the blood–brain-barrier and penetrate into NAc tissues.

### In the NAc, OCA treatment inhibits the ability of cocaine to augment electrically stimulated DA release

**Figure 2:**
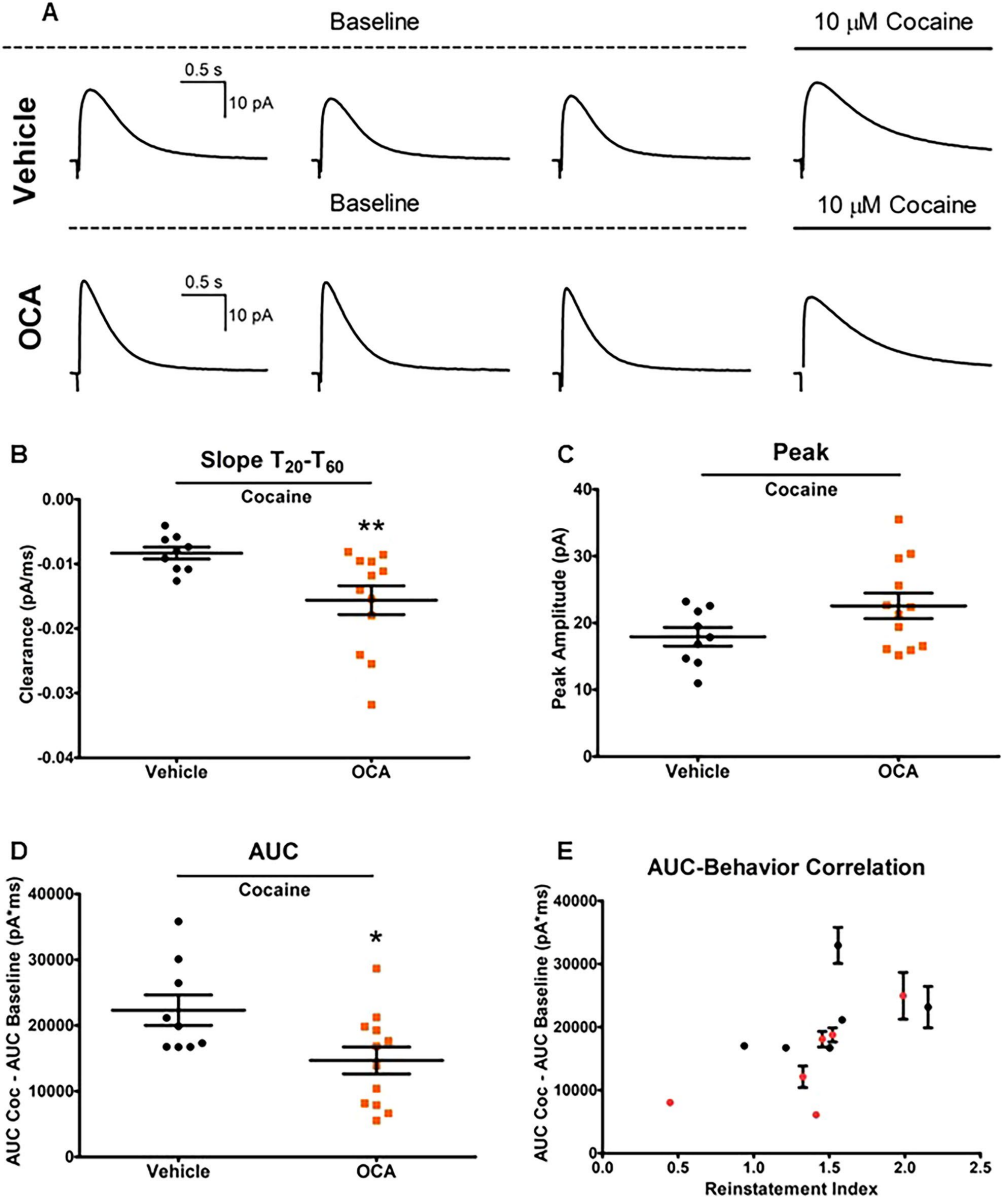
OCA treatment alters DA dynamics in the nucleus accumbens in response to cocaine. After completion of behavioral testing as in Fig. 1, mice treated with either vehicle or OCA were sacrificed and coronal brain slices containing the NAc were prepared for amperometric recording of electrically stimulated DA release. Data from vehicle and OCA treated mice are shown in black and red, respectively. (**A**) Representative amperometric traces obtained from animals treated with either vehicle or OCA in the absence (baseline) or presence of 10 µM cocaine. (**B**) Clearance rate for animals treated as in A, in the presence of 10 µM cocaine, calculated as the slope of the curve from 20% of the curve time (T_20_) to 60% of the curve time (T_60_) (two-tailed Student’s *t* test with Welch’s correction, *p* = 0.009, t = 3.03; n = 9 vehicle, n = 12 OCA). (**C**) Peak amplitude of the amperometric current obtained from animal, treated as in A, in the presence of 10 µM cocaine (two-tailed Student’s *t* test, *p* = 0.08, t = 1.84; n = 9 vehicle, n = 12 OCA). (**D**) Net AUC of amperometric traces of animals, treated as in A, calculated by subtracting the mean values of AUC at respective baselines from AUC in the presence of 10 µM cocaine (two-tailed Student’s *t* test, *p* = 0.024, t = 2.46, n = 9 vehicle, n = 12 OCA). (**E**) Correlation of net AUC and Reinstatement Index (Fig. 1E) for each animal (Pearson correlation coefficient: r = 0.60, *p* = 0.039, R^2^ 0.36). For animals in which 2 slices were used for recording, the average net AUC value was used.

Considering that cocaine-induced behaviors are associated with the ability of cocaine to elevate extracellular DA levels in the nucleus accumbens (NAc)^26,31,32^, we next determined whether OCA inhibits this action of cocaine. After completion of behavioral testing as in Fig. 1, mice treated with either vehicle or OCA were sacrificed, and coronal brain slices containing the NAc were prepared for amperometric recording of electrically stimulated DA release^7^. No significant differences were observed in DA release under baseline conditions (Fig. 2A, Supplemental Fig. 2). However, upon perfusion of 10 µM cocaine, significant differences were observed in the slope and the area under the curve (AUC) of the amperometric signals obtained from animals treated either with vehicle or OCA (Fig. 2). Slices from animals treated with OCA had faster DA clearance following electrical stimulation (-0.01561 ± 0.002224 pA/ms) compared to animals treated with vehicle (− 0.008318 ± 0.0009211 pA/ms, Fig. 2B). No significant differences in peak amplitude in the presence of cocaine were detected between the two groups (Fig. 2C). However, the difference in clearance rates translated to a diminished AUC of the amperometric signal for animals treated with OCA (14,670 ± 2051 pA*ms) versus vehicle (22,320 ± 2315 pA*ms, Fig. 2D). This data supports the notion that OCA treatment reduces the ability of cocaine to increase electrically stimulated DA release. We also found a positive correlation between the AUC of the amperometric signal, recorded upon cocaine treatment, and the Reinstatement Index (Fig. 2E; Pearson r = 0.60, *p* = 0.039, R^2^ 0.36). This data suggests that the potency of cocaine to cause reinstatement is dictated by its ability to raise extracellular DA levels.

### OCA treatment does not inhibit appetitive learning

**Figure 3:**
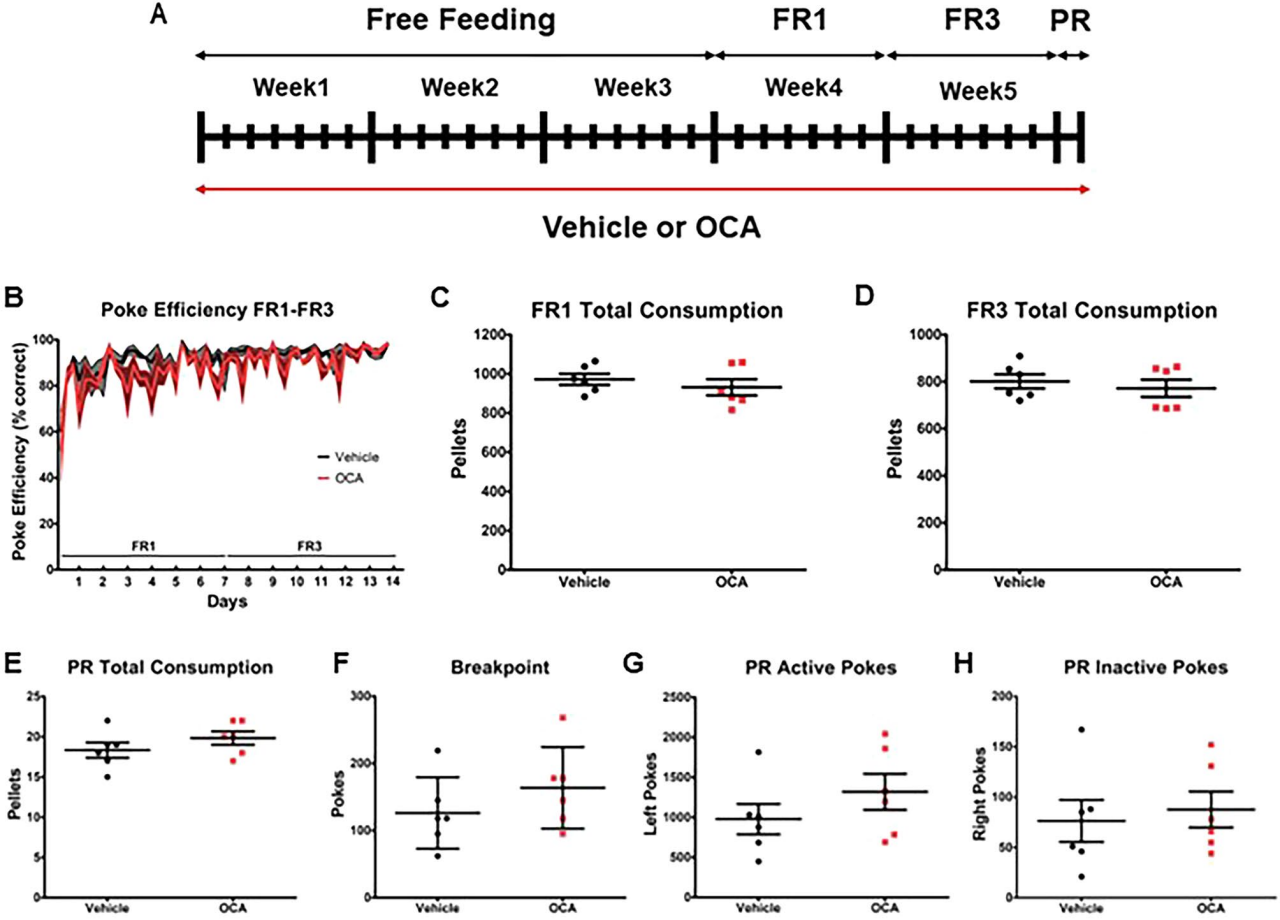
OCA treatment does not inhibit appetitive learning. (**A**) Schematic of study design. Mice were treated with either vehicle or 10 mg/kg OCA throughout the duration of the study. (**B**) Poke Efficiency (ratio of Active Pokes/Total Pokes, expressed as %) for Fixed Ratio 1 (FR1; RM Two-Way ANOVA, Treatment F(1,270) = 4.55, *p* = 0.059; Time F(27,270) = 5.24, p < 0.0001; Interaction F(27,270) = 0.75, *p* = 0.82) and Fixed Ratio 3 (FR3; RM Two-Way ANOVA, Treatment F(1,260) = 3.1, *p* = 0.11; Time F(26,260) = 1.9, *p* = 0.0067; Interaction F(26,260) = 1.22, *p* = 0.22). (**C**) Total pellet consumed during the FR1 paradigm (two-tailed Student’s *t* test, *p* = 0.44, t = 0.81; n = 6). (**D**) Total pellet consumed during the FR3 paradigm (two-tailed Student’s *t* test, *p* = 0.55, t = 0.62; n = 6). (**E**) Total pellet consumed during the progressive ratio (PR) paradigm (PR; two-tailed Student’s *t* test, *p* = 0.26, t = 1.18; n = 6). (**F**) PR breakpoint calculated as described in the Methods section (two-tailed Student’s *t* test, *p* = 0.28, t = 1.14; n = 6). (**G**) Total number of active pokes during the PR paradigm (two-tailed Student’s *t* test, *p* = 0.27, t = 1.16; n = 6). (**H**) Total number of inactive pokes during the PR paradigm (two-tailed Student’s *t* test, *p* = 0.69, t = 0.41; n = 6).

We next determined whether OCA, in addition to inhibiting both formation of CPP for cocaine^7^ as well as reinstatement of cocaine CPP, is also impairing contextual learning and/or motivation for natural rewards. To do this, mice were singly housed with an operant Feeding Experimentation Device (FED3). The FED3 features a pellet (regular chow) dispenser and two ‘nose-poke’ sensors. Only one of the two nose-poke sensors is linked to distribution of pellets, i.e. is “active”. In the paradigms in which the mouse is required to nose-poke to obtain food (FR1, FR3, PR), when the mouse pokes the active port, the FED3 delivers food combined with an auditory tone (4 kHz for 0.3 s) and a visual stimulus (eight blue LEDs light)^28^. These devices monitor the amount of food pellets retrieved and allow multiple behavioral tasks to be performed using the same device, such as learning a task to obtain food, as well as increasing the amount of “work” required to obtain food. Monitoring was continuous, performed in their home cages, and lasted for 5 weeks. The animals were fed either vehicle or OCA (10 mg/kg) once per day in jellies for the duration of the experiment (Fig. 3A). For the first 3 weeks, animals were given ad libitum access to food (free feeding) to facilitate acclimation to the FED3 device as the primary source of food. Next, the FED3 devices were programmed to perform fixed ratio reinforcement paradigms that required operant nose pokes in order to obtain food. In Week 4, devices were set to dispense food at an FR1 program (1 correct poke = 1 food pellet; no reward for incorrect pokes). In Week 5, devices were set to dispense food at an FR3 program (3 correct pokes = 1 food pellet; no reward for incorrect pokes). At the beginning of testing, it is typical for animals to use up to 12 h to learn the task, then resume a consistent retrieval pattern^28^. The poke efficiency (ratio of correct pokes/total pokes) was 60% and 52% for vehicle and OCA, respectively, in the first 6 h of FR1. The poke efficiency increased to 92% and 89% within 12 h, and remained relatively stable throughout the remainder of FR1 and FR3 testing (Fig. 3B). No effects of OCA treatment were measured throughout the different paradigms; Repeated Measures (RM) Two-Way ANOVA highlighted an effect of time alone (FR1 Time F(27,270) = 5.24, *p* < 0.0001; FR3 Time F(26,260) = 1.9, *p* = 0.0067). The total number of pellets consumed between vehicle (972.2 ± 28.27) and OCA (931.5 ± 41.61) groups did not differ during the FR1 paradigm (Fig. 3C). Similarly, no significant differences in total number of pellets consumed were observed between mice treated with vehicle (801.2 ± 30.32) and OCA (771.5 ± 37.13, Fig. 3D) during the FR3 paradigm. These data demonstrate that OCA does not impair appetitive learning or consumption.

At the completion of the FR3 schedule, we switched animals to a PR paradigm which allows determination of the break point, or limit, to the amount of “work” that the mouse is willing to perform in order to obtain food. During the PR schedule, the first pellet requires one correct poke, then progresses to higher ratio levels of correct pokes per pellet, in a nonlinear fashion (see methods). Breakpoint is defined as the number of pokes in the last completed task within a 3-h threshold. No differences were detected between vehicle and OCA groups for the total number of pellets obtained (Vehicle = 126.2 ± 21.75, OCA = 163.7 ± 24.80, Fig. 3E) nor for the breakpoint (Vehicle = 18.33 ± 0.96, OCA = 19.83 ± 0.83, Fig. 3F). In addition, no differences were detected between vehicle and OCA treated animals for the total number of pokes on the active port (Vehicle = 977.3 ± 189.8, OCA = 1318 ± 224.4, Fig. 3G) or pokes on the inactive port (Vehicle = 76.33 ± 20.86, OCA = 87.67 ± 17.85, Fig. 3H), suggesting no effects of OCA on learning or memory for a food operant task.

## Discussion

Treatment with the BA OCA, as well as bariatric surgery, which elevates circulating levels of BAs, reduces CCP for cocaine in mice^7^. In the current study, we show that OCA treatment, in addition to regulating the expression of contextual preference for cocaine, also reduced drug priming-induced reinstatement for cocaine CPP. Noteworthy is that OCA was administered during the extinction phase, a post-cocaine abstinence period, suggesting OCA as a possible pharmacological treatment for cocaine relapse. Indeed, OCA is a drug (OCALIVA®, Intercept Pharmaceuticals), approved by the FDA in 2016, and currently incorporated in guidelines as a second-line treatment for primary biliary cholangitis^33^. It is also currently in a phase 3 clinical trial for its action toward non-alcoholic steatohepatitis (clinical trial identifier: NCT02548351). Furthermore, in our animal and experimental paradigm, OCA is effective after just a few days of treatment (1–4 days), indicating a fast-acting potential that is desirable for PUD therapeutics^34,35^.

As DA is critical for the formation of CPP and reinstatement, we next wanted to assess the effects of OCA on DA clearance in the NAc. Although at least 3 different families of monoamines are found in the NAc, DA is, by far, present at the highest levels in the region, constituting approximately 75% of total monoamines^7^. Serotonin (5-HT) is the second highest concentrated monoamine in the NAc, being present at less than a fifth of the level of DA. Norepinephrine (NE) is the third highest concentrated monoamine, with levels equivalent to approximately 13% of DA levels^36^. As we conducted experiments ex vivo, in slices containing NAc, and attempted to measure minute differences in clearance rates of monoamines, we chose amperometry as the most efficient technique to obtain information on monoamine dynamics. While it is possible that 5-HT or NE contribute to the signals we obtained, given the relative ratios of monoamines in this brain region^7,37^ and that DA in the NAc is critically involved in the reinforcing properties of cocaine^38^, we believe that the primary monoamine driving the changes generated by OCA is DA.

The lack of any effects of OCA on basal amperometric signals post electrical stimulation suggests there is no impact on vesicular release, but instead, specific action toward monoamine transporters (Supplemental Fig. 2). In fact, previous work, in oocytes heterologously expressing the DAT, has indicated that OCA has no effect on transport kinetics^5,6^. This agrees with our current data that demonstrates no effect of OCA on basal DA clearance rates (prior to cocaine treatment) in mouse NAc. Our data also demonstrate that OCA treatment reduces the ability of cocaine to impair the clearance of DA in the NAc (Fig. 2B). This phenomenon translates to a diminished AUC of the amperometric signal upon cocaine exposure (Fig. 2D). Of note is that the ability of a specific psychostimulant to decrease DA clearance, and therefore, increase extracellular DA levels, is associated with its psychomotor properties^39^. We also show that although administration of OCA inhibits cocaine-associated contextual preference as well as reinstatement, it did not accelerate time to extinction, further suggesting that OCA regulates only specific cocaine-associated behaviors.

How OCA could alter clearance rates in response to cocaine, but not under basal conditions, is an intriguing question. One possibility is that OCA inhibits Ras homologous (Rho) A (RhoA) activity trough TGR5 signaling. RhoA is a member of the Rho family and its activity and expression increase in the NAc in response to cocaine CPP paradigms^40^. Of note, TGR5 signaling promotes PKA-mediated phosphorylation of RhoA at Ser188 (pS188RhoA)^41^, thereby inhibiting its function. Thus, it is possible that OCA, by activating TGR5 signaling, inhibits RhoA and, as a consequence, impairs the reinforcing properties of cocaine.

Considering that changes in circulating BAs have been associated with consumption of highly palatable food^24,42^, we next determined whether OCA inhibits consumption of food in animals on a regular chow. We demonstrate that on a regular chow diet, exposure to OCA did not alter food intake, motivation for “working” for food, or learning and memory for a food operant task, suggesting that OCA inhibition of cocaine reinstatement is not a generalized inhibition of reward.

### Supplementary Information

Supplementary Figures.

## Data Availability

The datasets generated during and/or analyzed during the current study are available from the corresponding author on reasonable request.
