# Supplementary material for: Bile acids modulate reinstatement of cocaine conditioned place preference and accumbal dopamine dynamics without compromising appetitive learning
Source: Sci Rep. 2023 Aug 17;13:13359. doi: 10.1038/s41598-023-40456-3 (PMC10435481; doi:10.1038/s41598-023-40456-3)
Supplement: Supplementary file 1 — Supplementary Figures. [file 41598_2023_40456_MOESM1_ESM.docx]

**
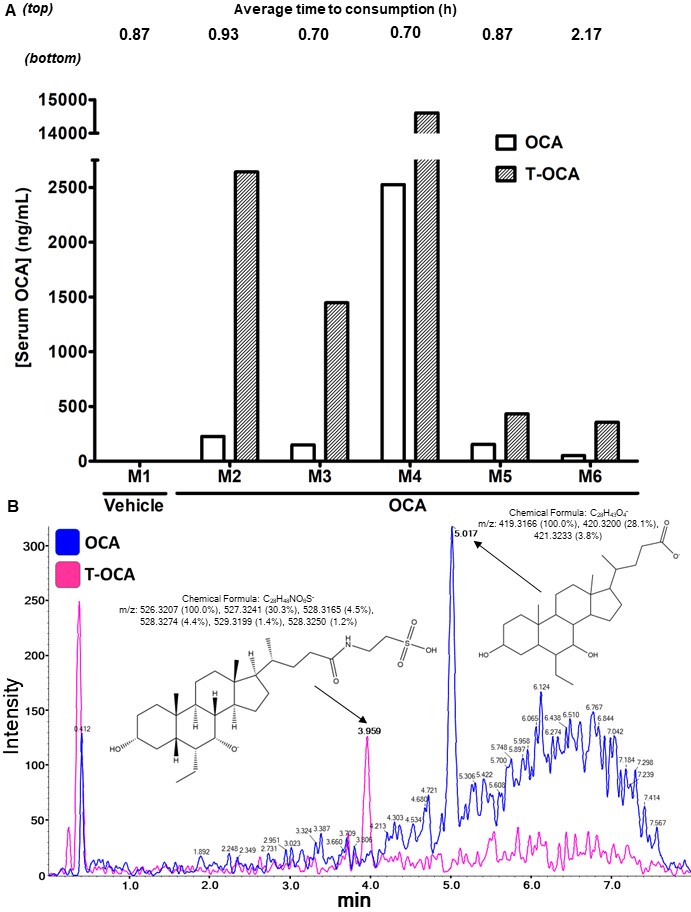
**

**Supplemental Figure 1. OCA administration through voluntary feeding results in detectable concentrations of OCA and T-OCA in the blood and brain of mice. (A)** Average time to jelly consumption over 3 days of feeding, (top). Unconjugated and Taurine-conjugated OCA (T-OCA) serum concentrations, in mice, after 4 feedings of vehicle or OCA (10 mg/kg) jellies, (bottom). **(B)** Representative extracted ion chromatograph of high-resolution negative ionization precursor scan masses of OCA (419.317 *m/z*) and T-OCA (526.321 *m/z*) from mouse 4 (M4) brain extract.


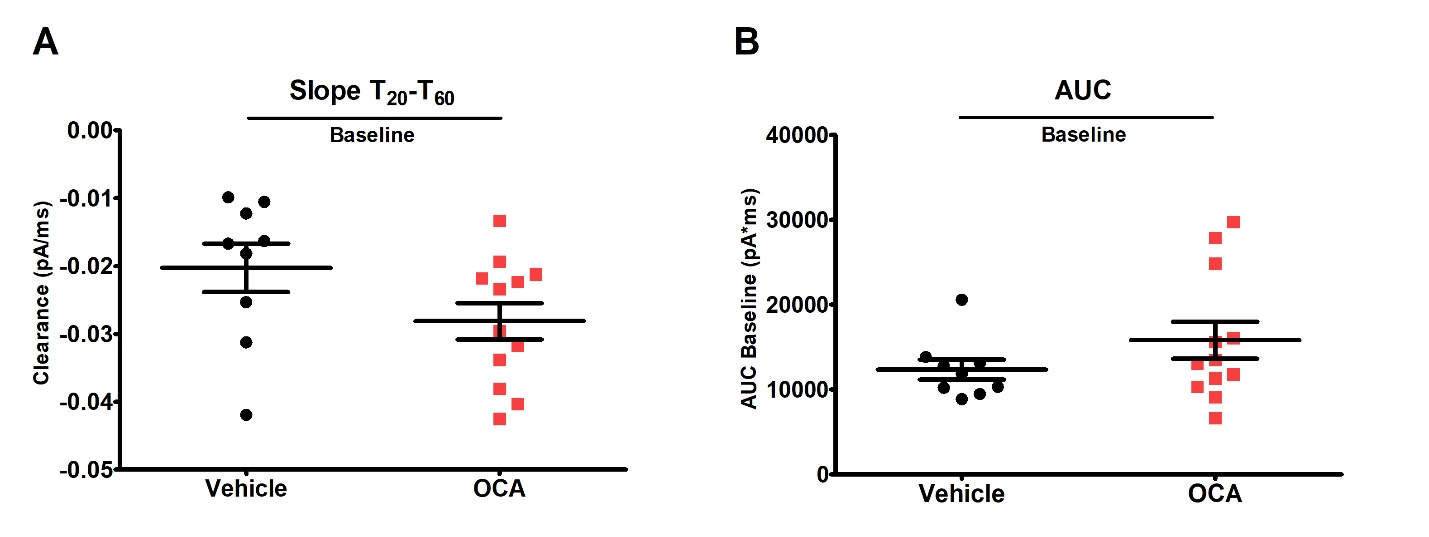


**Supplemental Figure 2. OCA treatment does not alter DA dynamics in the nucleus accumbens under baseline conditions. (A)** Clearance rates, of animals treated either with vehicle or OCA, as in figure 2, under baseline conditions (prior to application of 10 µM cocaine), calculated as the slope of the curve from 20% of the curve time (T_20_) to 60% of the curve time (T_60_). Vehicle = -0.02026 ± 0.003567, OCA = -0.02813 ± 0.002674 (two-tailed Student’s t-test, p= 0.09, t= 1.8; n= 9 vehicle, n=12 OCA). **(B)** AUC of amperometric signals, of animals treated with either vehicle or OCA, as in figure 2, under baseline conditions (prior to application of 10 µM cocaine). Vehicle = 12340 ± 1179, OCA = 15810 ± 2186 (two-tailed Student’s t-test with Welch’s correction, p= 0.18, t= 1.4, n= 9 vehicle, n= 12 OCA).
